# Inhibition of TGF-β1-induced epithelial-mesenchymal transition in gliomas by DMC-HA

**DOI:** 10.18632/aging.205340

**Published:** 2023-12-27

**Authors:** Lei Shi, Zhimin Wang, Jun Rong, Xifeng Fei, Xuetao Li, Bao He, Weiyi Gong, Jin Qian

**Affiliations:** 1 Department of Neurosurgery, Affiliated Kunshan Hospital of Jiangsu University, Suzhou 215300, P.R. China; 2 Department of Neurosurgery, Dushu Lake Hospital Affiliated to Soochow University, Suzhou 215300, P.R. China; 3 Department of Neurosurgery, Xuancheng People’s Hospital, The Affiliated Xuancheng Hospital of Wannan Medical College, Anhui 242099, P.R. China; 4 Department of Neurosurgery, Suzhou Kowloon Hospital, Shanghai Jiaotong University School of Medicine, Suzhou 215028, P.R. China

**Keywords:** DMC-HA, HDACs, TGF-β1, epithelial-mesenchymal transition, gliomas

## Abstract

DMC-HA, a novel HDAC inhibitor, has previously demonstrated antiproliferative activity against various cancers, including gliomas. However, the role of DMC-HA in the regulation of EMT and its underlying mechanisms remain unknown. This study aimed to explore the effects of DMC-HA on TGF-β1-induced EMT in human gliomas and the underlying mechanisms involved. Our results showed that TGF-β1 induced EMT of U87 and U251 cells, leading to a decrease in epithelial marker ZO-1 and an increase in mesenchymal markers N-cadherin and Vimentin. Moreover, TGF-β1 treatment resulted in a significant increase in the migratory and invasive abilities of the cells. However, treatment with DMC-HA effectively inhibited the augmented migration and invasion of glioma cells induced by TGF-β1. Additionally, DMC-HA inhibits TGF-β1-induced EMT by suppressing canonical Smad pathway and non-canonical TGF-β/Akt and Erk signalling pathways. These findings suggest that DMC-HA has potential therapeutic implications for gliomas by inhibiting EMT progression.

## INTRODUCTION

Gliomas are the most common primary intracranial tumors, with no definitive and effective treatment available, especially for malignant gliomas such as glioblastoma multiforme [1]. Due to their invasive growth into surrounding tissue, complete removal through surgery is challenging, and most patients experience recurrence after surgery with a poor prognosis [2].

Epithelial-mesenchymal transition (EMT) is a biological process where epithelial cells undergo transformation into mesenchymal phenotypes through specific programs [3]. This process leads to the loss of polarity, connection with the basement membrane, and cell-cell adhesion, ultimately resulting in the acquisition of properties such as high migration, invasion, anti-apoptosis, and extracellular matrix degradation, which are characteristics of mesenchymal cells [4]. EMT plays an important role in malignant tumor cells’ acquisition of the ability to migrate and invade and is a crucial step in tumor invasion and metastasis [5].

Recent studies suggest that EMT is a critical process involved in glioma invasion and migration [6]. The invasion and growth of glioma cells into surrounding tissues are related to the pathophysiological process of EMT [7]. The loss of tight junctions between cells during EMT leads to the transformation of epithelial cells into mesenchymal cells with high migration and invasion properties, which is a key initiating step for tumor invasion and metastasis [8]. Although GBM progression does not undergo complete EMT, it does undergo a transition process similar to EMT, termed epithelial-mesenchymal transition-like (EMT-like) [9]. This process is characterized by a state with a less epithelial phenotype and a more mesenchymal phenotype, mainly manifested by downregulation of epithelial markers such as E-cadherin and claudin 1 (ZO-1) expression and induction of mesenchymal markers such as N-cadherin, fibronectin (FN), Vimentin, and zinc finger E-box binding protein 1 (ZEB1) elevation, ultimately leading to the weakening of intercellular adhesion and loss of cell polarity, which ultimately enhances tumor invasion and migration [10]. Clinical samples have demonstrated that EMT is closely related to the invasion and growth of malignant gliomas.

TGF-β is considered the most important factor in inducing EMT during development, cancer, and other pathological conditions [11]. In some cultured epithelial cell lines, TGF-β stimulation alone can induce EMT. *In vitro* and *in vivo* experiments with GBM cell lines have shown that TGF-β has the potential to induce EMT. For example, TGF-β-treated U87 and U251 glioma cells undergo mesenchymal-like transformation through the induction of the Smad2 signaling pathway, leading to morphological changes, up-regulation of mesenchymal markers, and increased glioma cell invasion and migration [12, 13]. In this study, we investigated the effects of DMC-HA, a novel HDAC inhibitor, on TGF-β1-induced EMT in glioma cells. DMC-HA, as a curcumin derivative, is likely to share some of the properties and mechanisms of its parent compound [14]. Curcumin has been widely studied for its potential to inhibit EMT in various types of tumors, including gliomas, and its mechanisms have been shown to impact multiple signaling pathways, including Smad and AKT pathways [15–18]. Given this background, it’s reasonable to position the study as an exploration of DMC-HA’s potential in inhibiting glioma EMT and elucidating its underlying mechanisms.

## MATERIALS AND METHODS

### Cell line and culture conditions

Human glioblastoma cell lines U87 and U251 were purchased from Shanghai Chinese Academy of Sciences Cell Bank and cultured in Dulbecco’s modified Eagle’s medium (DMEM) with 10% fetal bovine serum and 1% penicillin/streptomycin. Then, cells were maintained in a humidified incubator containing 5% CO_2_ at 37°C. The medium was changed every two days and used for cell experiments when cell proliferation reached an exponential stage.

### Cell viability assay for cell proliferation

The cell viability of U87 and U251 cells were assessed by CCK-8 assay. 5 × 10^3^ cells of U87 and U251 were seeded into 96-well plates and treated with TGF-β1 and/or DMC-HA for indicated time. After adding CCK8 solution, the absorbance of each hole was measured at 450 nm a microplate reader (Bio-Rad, Hercules, CA, USA). Each independent experiment was repeated three times.

### Transwell assays for cell migration and invasion

Cell invasion and migration were assessed using Transwell assays with BD Transwell Chambers, which were either coated with Matrigel (for the cell invasion assay) or left uncoated (for the cell migration assay), both obtained from BD Biosciences, Franklin Lakes, NJ, USA. U87 and U251 cells were treated with TGF-β1 and/or DMC-HA for 24 h. Then, cells were trypsinized and seeded into the top chamber at a density of 5 × 10^4^ cells per well with serum-free DMEM. The bottom chamber was added DMEM containing 20% FBS at 37°C, 5% CO_2_ condition. After 24 h incubation, the cells on the top chamber were wiped away with cotton swabs, and then the cells on basement membrane were fixed with 4% paraformaldehyde and stained by crystal violet. Cells were counted at random six independent fields of each well under a microscope.

### Western blot analysis

3 × 10^5^ cells of U87 and U251 cells were seeded in 6-cm dishes and exposed to TGF-β1 and/or DMC-HA at indicated times. Cell lysate was extracted by using RIPA lysis buffer (Beyotime Biotechnology, Nanjing, China) and centrifuged at 12,000 rpm for 15 min. After protein quantification, 50 μg/lane protein was added to each pore, and were separated on SDS-PAGE (Beyotime Biotechnology, Nanjing, China) under 140 V condition for 1.5 h and transferred to PVDF membranes (Merck Millipore, Darmstadt, Germany) under 300 mA for 60 min. Then PVDF membranes were incubated with each primary antibody overnight at 4°C. Antibodies against ZO-1 (Rabbit pAb, 21773-1-AP, Proteintech, USA), N-cadherin (Rabbit pAb, 22018-1-AP, Proteintech), Vimentin (Rabbit pAb, 10366-1-AP, Proteintech), Snail (Rabbit mAb, 3879, CST, USA), Slug (Rabbit mAb, 9585, CST), Twist1 (Rabbit mAb, 69366, CST), MMP-2 (Rabbit pAb, 10373-2-AP, Proteintech), MMP-9 (Rabbit pAb, 27306-1-AP, Proteintech), p-Smad2 (Rabbit mAb, 18338, CST), Smad2 (Rabbit pAb, 12570-1-AP, Proteintech), p-Smad3 (Rabbit mAb, 9520, CST), and Smad3 (Mouse mAb, 66516-1-Ig, Proteintech) were indicated dilution rate according to their instructions, respectively. Anti-GAPDH antibody (Mouse mAb, 60004-1-Ig, Proteintech) was used to check for equal loading. After incubating with corresponding secondary antibodies, the PVDF membranes were detected with and enhanced chemiluminescence (Amersham Life Science, Arlington Heights, IL, USA).

### *In vivo* study

Six-week-old BALB/c nude mice were obtained from the Shanghai Experimental Animal Center of the Chinese Academy of Sciences (Shanghai, China). U87 cells, totaling 5 × 10^6^, were pre-treated with TGF-β1 (10 ng/mL) and then subcutaneously implanted into the right flank of BALB/c nude mice. Once the xenograft tumors reached approximately 100 mm^3^ in size, the mice were randomly divided into two groups: DMSO+TGF-β1 and TGF-β1+DMC-HA. Subsequently, the mice received intraperitoneal injections of 20 mg/kg DMC-HA once daily over the course of two weeks. After this period, the nude mice were humanely euthanized, and the tumors were harvested for further examination.

### Histoscore analysis

In immunohistochemistry (IHC), the expression of MMP-2, MMP-9, N-cadherin, and Vimentin was semi-quantified based on two parameters: staining intensity, graded on a scale from 0 to 3 (0 = negative, 1 = weak, 2 = moderate, and 3 = strong), and the percentage of tumor cells stained, assessed on a scale from 0 to 4 (0 = <1%, 1 = 1–25%, 2 = 26–50%, 3 = 51–75%, and 4 = ≥75% of tumor cells). The histoscore was calculated by multiplying the staining intensity by the percentage of immunopositive tumor cells, resulting in scores ranging from 0 to 12. A histoscore of 1–3/12 was categorized as low expression, while a score of ≥4/12 was categorized as high expression [19].

### Statistical analyses

Statistical analyses were performed using SPSS 21.0 software (SPSS, Chicago, IL, USA). The results were expressed as means ± standard deviations (SD). The difference between the groups was determined using ANOVA with repeated measures. To compare the difference between the two groups, the independent sample *t* test was used. Statistical significance was considered at the level of ^*^p < 0.05.

## RESULTS

### TGF-β1 induces EMT of U87 and U251 cells

**Figure 1:**
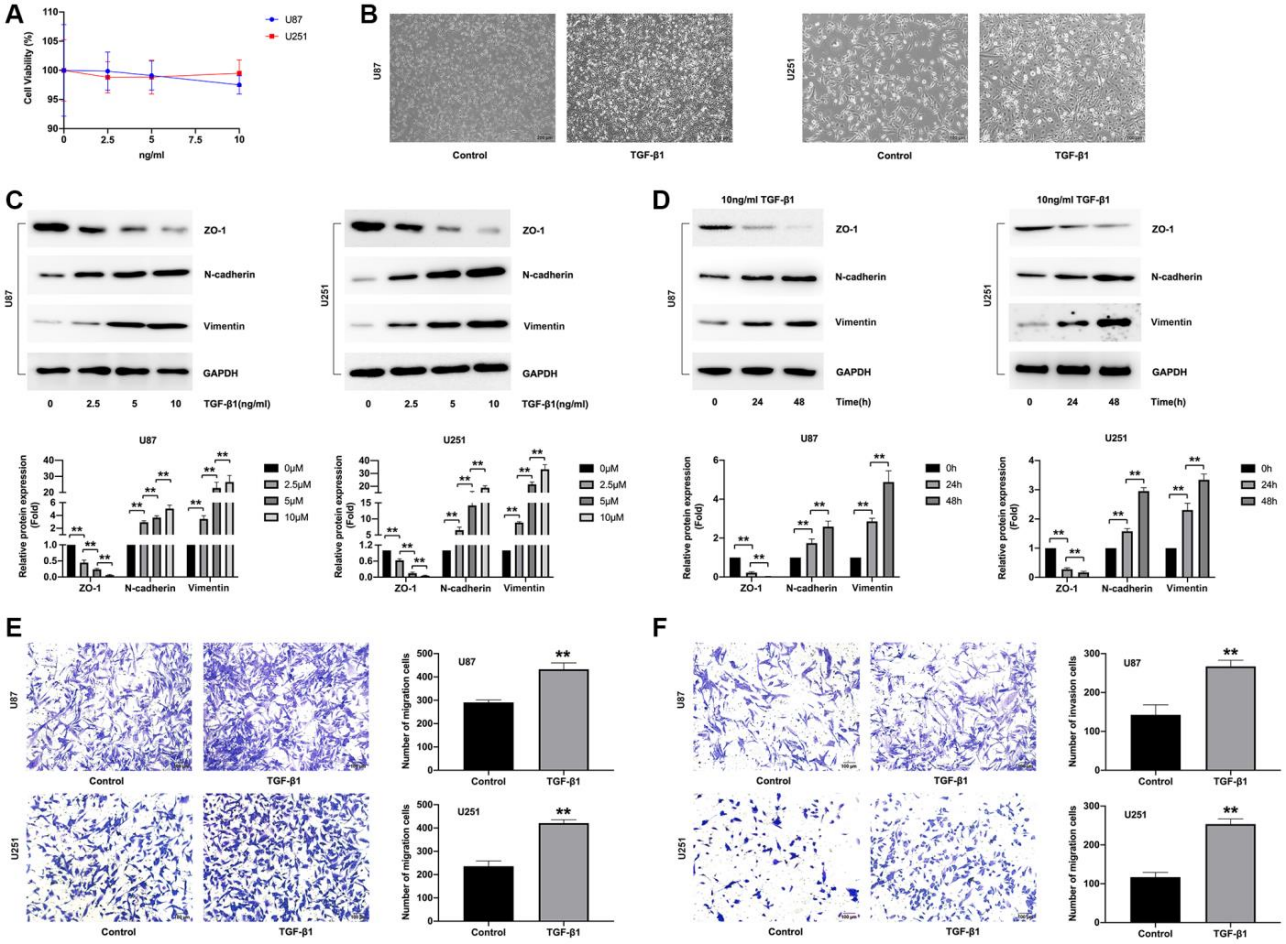
**Effects of TGF-β1 on the EMT of glioma cells.** (**A**) U87 and U251 cells were treated with the indicated concentrations of TGF-β1 (0, 2.5, 5 and 10 ng/mL) for 48 h, and then cell viability was detected using CCK-8 assay. (**B**) Effects of 10 ng/mL TGF-β1 on morphology of U87 and U251 cells. (**C**, **D**) U87 and U251 cells were treated with 10 ng/mL TGF-β1 for 48 h, and then the expression of ZO-1, N-cadherin and Vimentin proteins were detected by Western blot analyses. (**E**) Assessment of U87 and U251 cell invasion in response to 10 ng/mL TGF-β1 used a light microscope following Matrigel migration assay staining. (**F**) Assessment of U87 and U251 cell invasion in response to 10 ng/mL TGF-β1 used a light microscope following Matrigel invasion assay staining.

To assess whether DMC-HA repressed TGF-β1-induced EMT in gliomas, we first check the effects of TGF-β1 on inducing EMT of U87 and U251 cells. To rule out the effect of proliferation on EMT, U87 and U251 cells were treated with a range concentration of TGF-β1 and then cell proliferation ability was assessed by CCK-8 assay. As shown in Figure 1A, no obvious change of cell viability was observed in U87 and U251 cells after TGF-β1 below 10 ng/mL treatment for 48 h. Further, morphological changes of TGF-β1-induced EMT were evaluated in U87 and U251 cells. As shown in Figure 1B, star-like morphology of U87 and U251 cells were changed to a spindle-like, stretched and elongated shape and the typical features of mesenchymal cells.

Further, we analyzed the effects of TGF-β1 on EMT markers expression including ZO-1, N-cadherin and Vimentin in U87 and U251 cells. ZO-1 is one of epithelioid cell specific markers, while N-cadherin and Vimentin are mesenchymal-like cell-specific markers. Our results showed that TGF-β1 decreased the expression of ZO-1 and increased the expression of N-cadherin and Vimentin in a dose dependent (Figure 1C) and time dependent (Figure 1D). The phenomenon of the down-regulation of epithelial marker ZO-1 and the concomitant elevation of mesenchymal markers such as N-cadherin and Vimentin suggested that the induction of TGF-β1 enables GBM cells to undergo epithelial-mesenchymal transition [20–22]. During EMT, epithelial cell polarity is lost, contacts with surrounding cells and stromal cells are reduced, cell-to-cell interactions are reduced, and cell migration and motility are enhanced [23]. Accordingly, the migratory and invasive abilities of the cells were further evaluated after TGF-β1 treatment. As shown in Figure 1E, 1F, cell migration and invasion of U87 and U251 cells were significantly increased after 10 ng/mL TGF-β1 treatment.

### DMC-HA inhibits TGF-β1-induced migration and invasion of glioma cells *in vitro*

**Figure 2:**
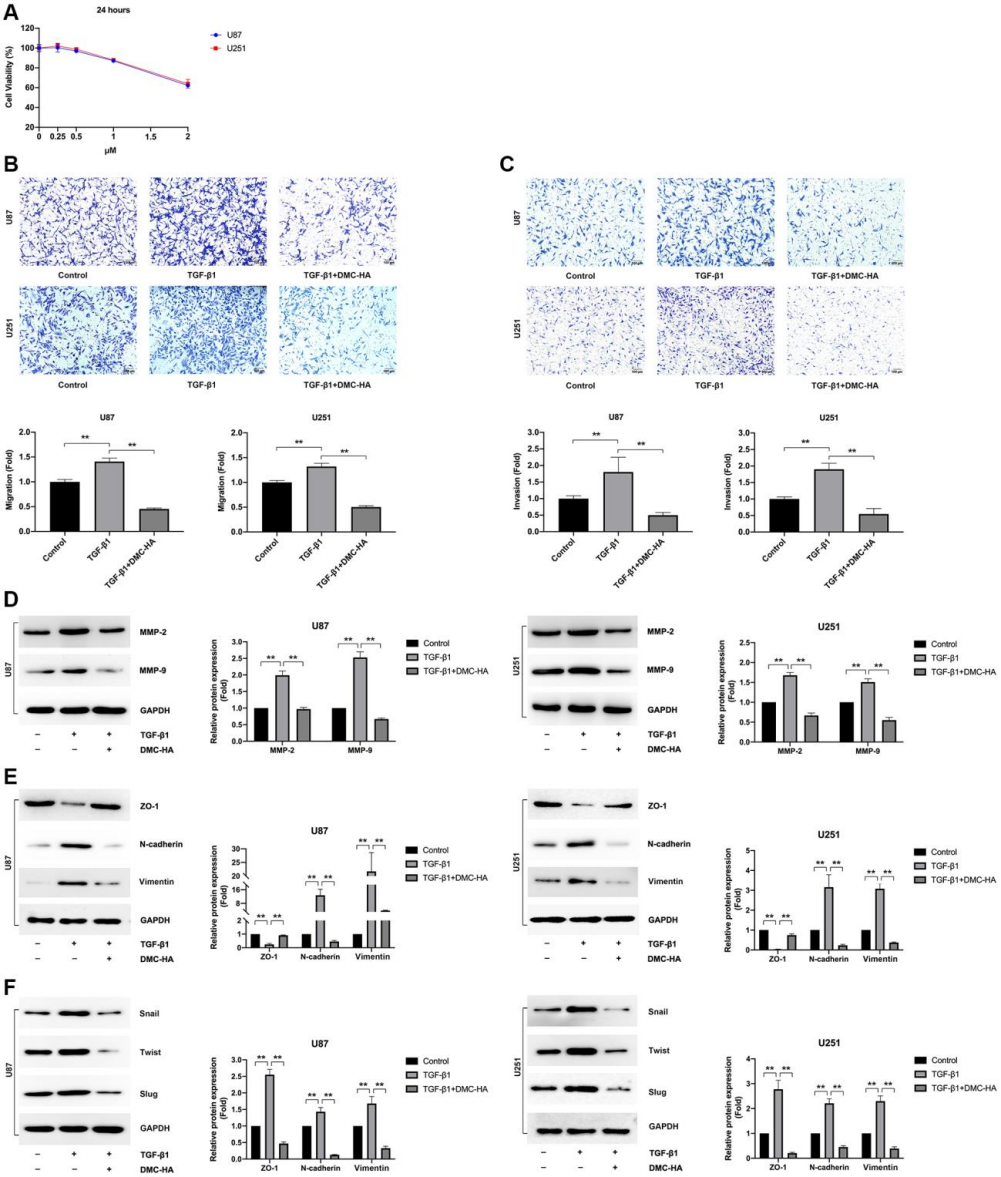
**Effects of DMC-HA on the migration and invasion of glioma cells.** (**A**) Cell viability in U87 and U251 cells was assessed using the CCK-8 assay following the indicated DMC-HA treatments. (**B**, **C**) Transwell assays were employed to evaluate the migration and invasion of U87 and U251 cells treated with 1 μM DMC-HA. (**D**) Western blot analysis was utilized to examine the expression of MMP-2 and MMP-9 proteins following a 48-hour treatment with DMC-HA and TGF-β1. (**E**) Western blot analysis was performed to assess the expression of ZO-1, N-cadherin, and Vimentin proteins after a 48-hour treatment with DMC-HA and TGF-β1. (**F**) Western blot analysis was used to detect the expression of Snail, Twist, and Slug proteins after a 48-hour treatment with DMC-HA and TGF-β1.

DMC-HA is an HDAC inhibitor that has been demonstrated to modulate EMT progression and inhibit migration and invasion in various types of cancers [24]. To evaluate the effects of DMC-HA on TGF-β1-induced glioma EMT-associated migration and invasion, we first conducted CCK-8 assays to exclude any potential effects of DMC-HA on cell proliferation. Our data showed that DMC-HA had negligible effects on cell viability at concentrations below 1 μM (Figure 2A), prompting us to use a concentration of 1 μM for the subsequent experiments. As depicted in Figure 2, treatment with DMC-HA effectively curtailed the augmented migration (Figure 2B) and invasion (Figure 2C) of glioma cells induced by 10 ng/mL TGF-β1. Furthermore, we examined the expression of EMT-associated proteins MMP-2 and MMP-9 and found that DMC-HA treatment significantly inhibited their increased expression induced by TGF-β1 (Figure 2D).

To further substantiate the inhibitory effects of DMC-HA on glioma cell EMT, we examined the changes in EMT-related proteins following treatment with TGF-β1 and/or DMC-HA. Our data indicated that DMC-HA reinstated the reduced levels of ZO-1 and impeded the upregulation of N-cadherin and Vimentin (Figure 2E) induced by 10 ng/mL TGF-β1. Moreover, DMC-HA suppressed the EMT-related transcriptional repressors Snail, Twist, and Slug, whose expression was also elevated during TGF-β1-induced EMT (Figure 2F). These findings suggest that DMC-HA effectively inhibits TGF-β1-induced glioma EMT by modulating EMT-related proteins.

### Effects of DMC-HA on the canonical Smad pathway of TGF-β1-induced EMT in glioma cells

**Figure 3:**
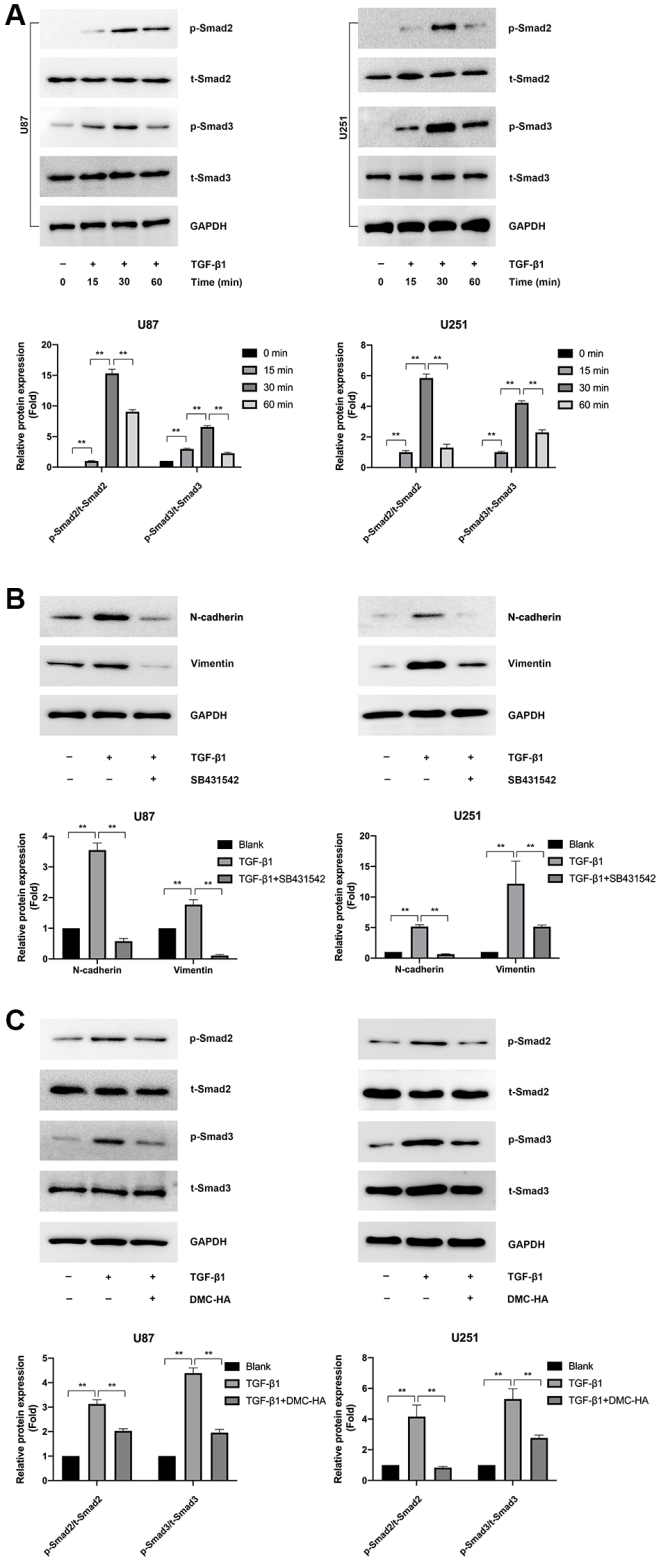
**Effects of DMC-HA on the Smad pathway in glioma cells.** (**A**) Western blot analysis was conducted to assess the expression of p-Smad2 and p-Smad3 after a 60-minute treatment with TGF-β1. (**B**) Western blot analysis was employed to examine the expression of N-cadherin and Vimentin following combined treatment with TGF-β1 and SB431542. (**C**) Western blot analysis was performed to evaluate the phosphorylation of Smad2 and Smad3 after co-treatment with TGF-β1 and DMC-HA.

TGF-β signaling-mediated EMT is commonly facilitated through the canonical Smad pathway [25]. In this pathway, TGF-β signaling activates Smad2 and Smad3 via a tetrameric complex comprising type I and type II receptors (TbRI and TbRII) and subsequently binds to Smad4. These Smad complexes translocate to the nucleus, where they cooperate with transcription factors to modulate the repression or activation of target genes. We first treated U87 and U251 cells with 10 ng/mL TGF-β1 for 60 min, followed by assessing the phosphorylation status of Smad2 and Smad3 (p-Smad2 and p-Smad3) using Western blot analysis. As depicted in Figure 3A, TGF-β1 substantially increased p-Smad2 and p-Smad3 expression in a time-dependent manner, reaching a peak at 30 min. Furthermore, TGF-β1-induced EMT effects were markedly alleviated by treatment with 1 μM SB431542 (a TGF-β1 inhibitor) (Figure 3B).

We subsequently examined whether DMC-HA mitigated TGF-β1-induced EMT via inactivation of the TGF-β1-Smad2/3 signaling pathway. U87 and U251 cells were pre-treated with 1 μM DMC-HA for 60 min, followed by exposure to 10 ng/mL TGF-β1 for 30 min. As illustrated in Figure 3C, DMC-HA significantly diminished TGF-β1-induced phosphorylation of Smad2 and Smad3 in both U87 and U251 cells. These findings suggest that DMC-HA inhibits TGF-β1-induced EMT by suppressing Smad2 and Smad3 phosphorylation.

### Effects of DMC-HA on the non-canonical TGF-β/Akt and Erk signalling pathways of TGF-β1-induced EMT in glioma cells

**Figure 4:**
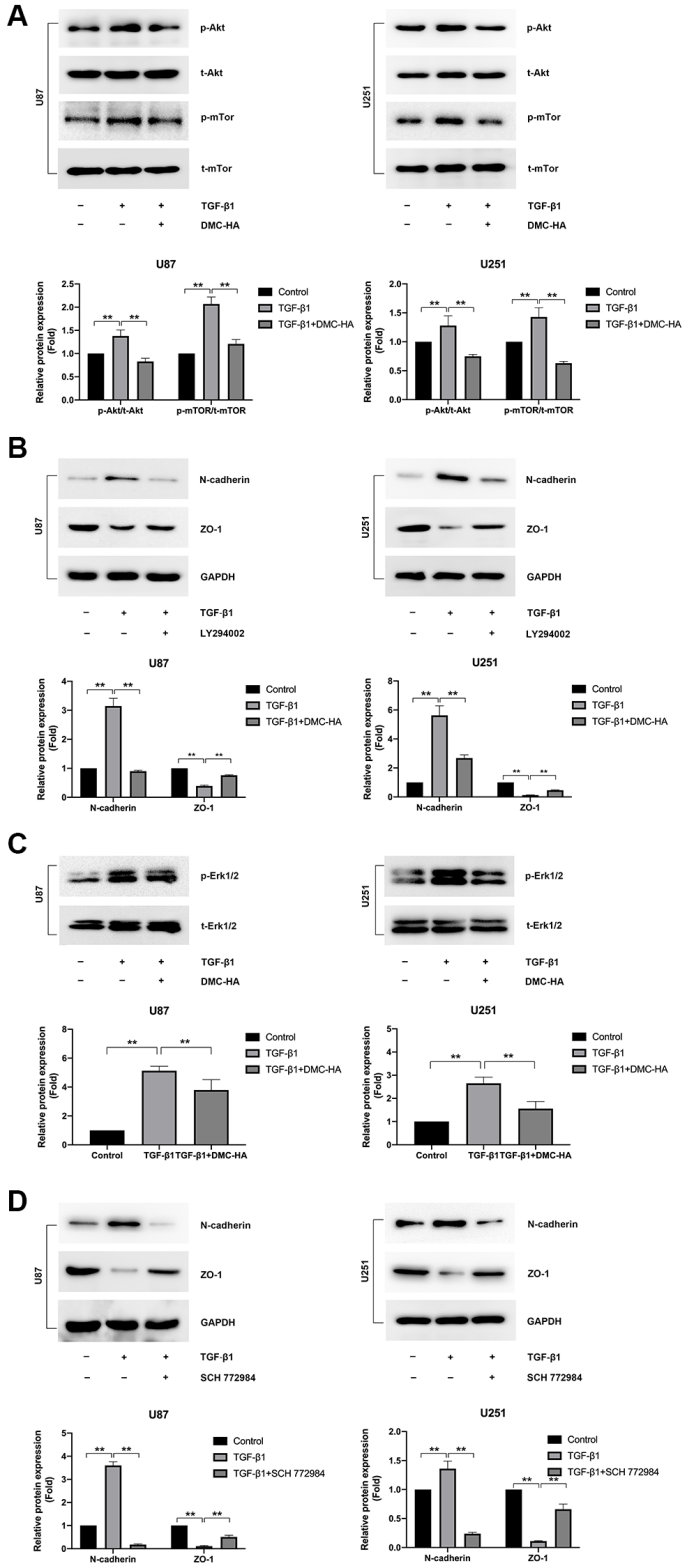
**Effects of DMC-HA on the Akt and Erk signalling in glioma cells.** (**A**) Western blot analysis was conducted to assess the expression of p-Akt and p-mTor following combined treatment with TGF-β1 and DMC-HA. (**B**) Western blot analysis was conducted to assess the expression of N-cadherin and ZO-1 following combined treatment with TGF-β1 and LY294002. (**C**) Western blot analysis was conducted to assess the expression of p-Erk1/2 following combined treatment with TGF-β1 and DMC-HA. (**D**) Western blot analysis was conducted to assess the expression of N-cadherin and ZO-1 following combined treatment with TGF-β1 and SCH 772984.

In addition to the canonical Smad pathway, the PI3K/AKT and Erk pathways are also commonly implicated in the process of TGF-β1-induced EMT [20–26]. We first examined the effect of DMC-HA on TGF-β1–induced activation of the PI3K/AKT pathway in U87 and U251 cells, which were pretreated with 1 μM DMC-HA for 60 min and then stimulated with 10 ng/mL of TGF-β1 for 48 hours. As depicted in Figure 4A, TGF-β1 induced phosphorylation of Akt and mTor in both U87 and U251 cells *in vitro*; however, co-treatment with DMC-HA and TGF-β1 significantly abrogated TGF-β1-induced Akt and mTor phosphorylation. To further explore the role of PI3K/AKT in TGF-β1-induced EMT, we employed the PI3K/AKT inhibitor LY294002 (5 μM) to treat U87 and U251 cells for 60 min and then stimulated with 10 ng/mL of TGF-β1 for 48 hours. As anticipated, LY294002-treated cells were not susceptible to TGF-β1-induced EMT (Figure 4B).

We next investigated the effects of DMC-HA on Erk activation during DMC-HA-mediated TGF-β1-induced EMT. As illustrated in Figure 4C, TGF-β1 treatment noticeably increased the phosphorylation level of Erk1/2, while co-treatment with DMC-HA considerably dampened TGF-β1–induced phosphorylation of Erk1/2. The specific Erk1/2 inhibitor, SCH 772984, markedly impeded the process of TGF-β1-induced EMT (Figure 4D). Collectively, these findings suggest that the inactivation of PI3K/AKT and Erk by DMC-HA contributes to the attenuation of EMT in glioma cells.

### Effect of DMC-HA on EMT-related genes *in vivo*

**Figure 5:**
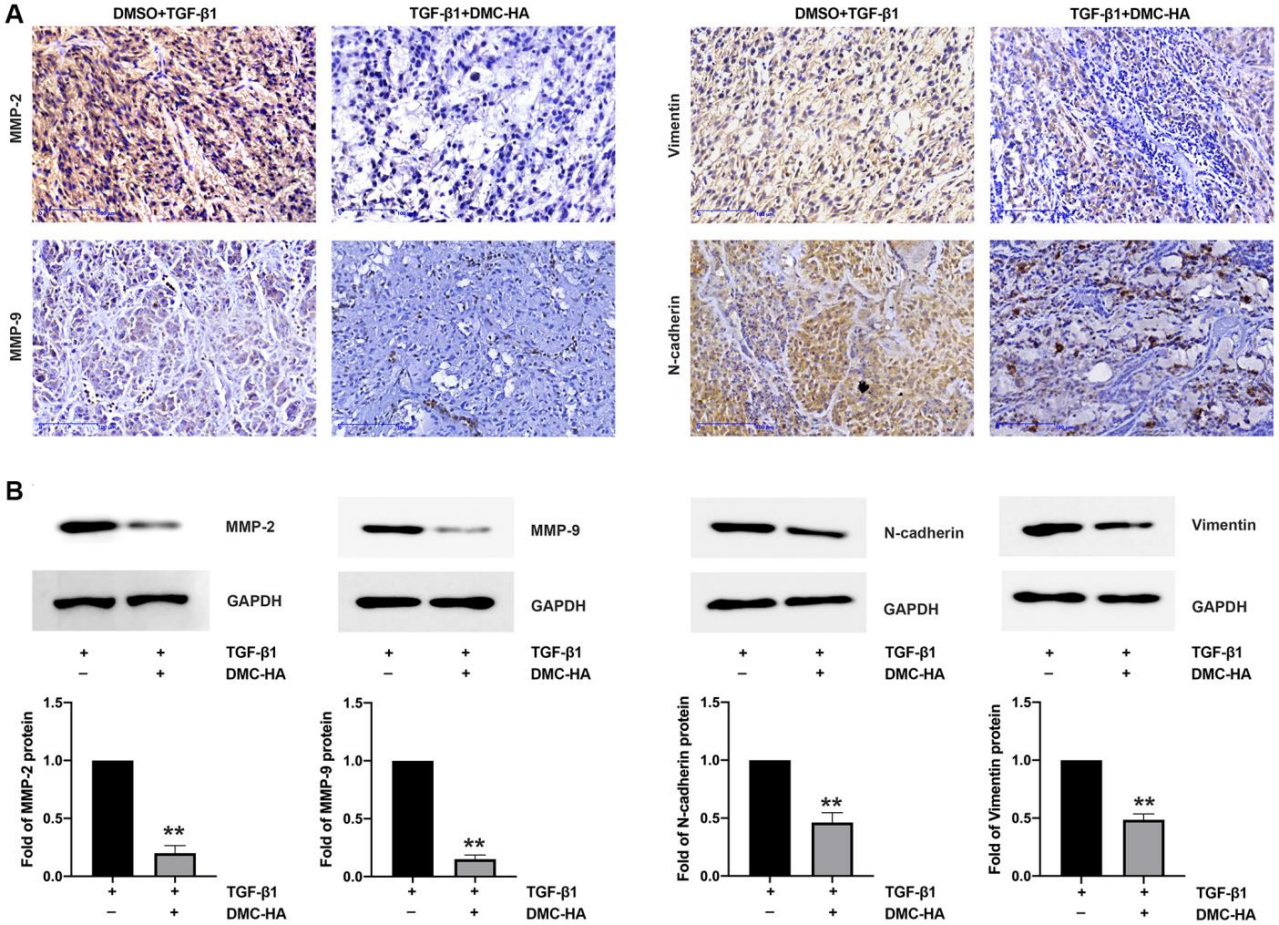
**DMC-HA inhibited TGF-β1 induced EMT *in vivo*.** (**A**) Immunohistochemical (IHC) staining was performed to evaluate the expression of MMP-2, MMP-9, N-cadherin, and Vimentin in tumor tissues treated with DMC-HA and/or TGF-β1. (**B**) Western blot analysis was carried out to examine the expression levels of MMP-2, MMP-9, N-cadherin, and Vimentin in tumor tissues treated with DMC-HA and/or TGF-β1.

To further validate the *in vitro* effects of DMC-HA on EMT, we utilized a TGF-β1-induced U87 xenograft model to evaluate its inhibitory effects on EMT *in vivo*. After two weeks of treatment with DMC-HA, we examined the levels of MMP-2, MMP-9, N-cadherin, and Vimentin using both immunohistochemistry (IHC) and Western blot assays. As shown in Figure 5A, the IHC staining revealed a significant decrease in the expression of MMP-2, MMP-9, N-cadherin, and Vimentin in DMC-HA-treated tumor tissues. The histoscore analysis revealed that xenograft tumors induced by TGF-β1 displayed high histoscores for MMP-2, MMP-9, N-cadherin, and Vimentin (>4/12). In contrast, xenograft tumors treated with both TGF-β1 and DMC-HA displayed low histoscores for MMP-2, MMP-9, N-cadherin, and Vimentin (<4/12). This trend was further confirmed by Western blot analysis, as shown in Figure 5B. Collectively, these findings provide additional evidence for the inhibitory effects of DMC-HA on EMT *in vivo*.

## DISCUSSION

Invasive growth is a prominent characteristic of GBM cells, which is characterized by their malignant infiltration into the surrounding tissues, rendering them difficult to be completely removed during surgery [27]. Consequently, most patients suffer from relapse after surgery, leading to a poor prognosis. Recent studies have revealed that the invasive growth and proliferation of GBM cells into surrounding tissues may be attributed to the pathophysiological process of epithelial-mesenchymal transition (EMT) [28]. EMT has been extensively studied in epithelial tumors, where the loss of tight junctions between cells and transformation into mesenchymal cells with high migratory and invasive potential are the initial steps of tumor invasion and metastasis. Additionally, programmed activation of EMT enables tumor cells to acquire unique metastatic capabilities to generate circulating tumor cells (CTCs) [29]. Subsequently, after the circulation of CTCs through the bloodstream, mesenchymal-epithelial transition (MET) occurs, leading to the formation of secondary metastatic lesions and the generation of new tumors in another part of the body. Therefore, EMT plays a crucial role in the invasive growth and metastasis of GBM cells.

However, the occurrence of EMT in malignant glioma has been a topic of debate, mainly due to the lack of typical epithelial cell characteristics and low expression of E-cadherin in most gliomas [30]. However, recent studies have shown that the typical E-cadherin to N-cadherin transition during the EMT process is not a prerequisite for promoting the invasive mesenchymal cell phenotype of GBM. Pala et al. have provided evidence supporting this finding [31]. Additionally, interstitial changes in GBM have been closely linked to its rapidly evolving clinical phenotype, further suggesting that the EMT process is closely associated with the invasion performance of GBM [32]. Moreover, bioinformatic analysis has revealed that most GBM samples exhibit more mesenchymal than epithelial features in terms of their epithelial and mesenchymal phenotypes. If induced, this phenotype can shift towards a more mesenchymal phenotype during an EMT-like process in glioma cells, further confirming the presence of EMT in GBM [33].

TGF-β signaling is considered the most critical factor in inducing EMT, and TGF-β is widely used as a tool to induce EMT in cells [34]. In this study, we also utilized TGF-β1 to induce EMT in glioma cells. We observed that under the induction of TGF-β1, glioma cells U87 and U251 exhibited typical mesenchymal cell morphology changes. Further analysis of critical markers after EMT activation demonstrated a significant increase in mesenchymal cell-specific markers such as N-cadherin and Vimentin, while epithelial cell-specific markers such as ZO-1 decreased significantly. Moreover, the induction of TGF-β1 significantly enhanced the migration and invasion ability of cells, indicating the activation of EMT in glioma cells.

In this study, we explored the effects of DMC-HA on TGF-β1-induced migration and invasion of GBM cells. Our results demonstrated that DMC-HA effectively inhibited the migration and invasion of GBM cells, accompanied by a decrease in the expression of MMP-2 and MMP-9. It is well-established that cell migration and invasion are complex processes closely associated with EMT, which is a crucial event in cancer progression. EMT allows cells to acquire a mesenchymal phenotype and enhanced migratory capacity, ultimately promoting cell migration and invasion.

Curcumin has been reported to inhibit EMT-related transcription factors in hepatocellular carcinoma and downregulate mesenchymal markers in breast cancer cells [35, 36]. Considering DMC-HA as a derivative of curcumin, we investigated its effects on EMT-related markers. Our data showed that DMC-HA can recover the protein level of the epithelial marker ZO-1 and decrease the levels of mesenchymal markers N-cadherin and vimentin induced by TGF-β1. Our *in vivo* experiments further confirmed this effect. Taken together, our study indicates that DMC-HA has the potential to inhibit cell migration and invasion in GBM cells, possibly by inhibiting the EMT process. This highlights the therapeutic potential of DMC-HA in treating GBM and warrants further investigation in preclinical and clinical studies.

However, the mechanism by which DMC-HA inhibits EMT in GBM cells was not previously known. EMT can be regulated by several signaling pathways, including classic and non-classical pathways. The classic pathway of EMT involves the activation of the transforming growth factor-beta (TGF-β) signaling pathway, which leads to the activation of transcription factors such as Snail, Slug, and Twist, and can directly or indirectly repress the expression of epithelial genes and activate the expression of mesenchymal genes [37]. In addition to the classic pathway, there is also a non-classical pathway of EMT that is induced by various stimuli such as growth factors, hypoxia, and metabolic changes. In the non-classical pathway, EMT is initiated by the activation of specific signaling pathways such as PI3K/AKT, ERK/MAPK, and JAK/STAT [38]. Surprisingly, the results of our study show that DMC-HA has the potential to inhibit epithelial-mesenchymal transition (EMT) in glioblastoma cells through multiple signaling pathways. In addition to the canonical Smad pathway, which has been previously implicated in EMT, we found that DMC-HA can also inhibit EMT through non-canonical pathways such as PI3K/AKT and Erk pathways. The PI3K/AKT pathway is known to play a role in cell growth, survival, and migration, and has been implicated in cancer progression. Our data suggest that DMC-HA inhibited this pathway, which might contribute to its anti-cancer effects; while PI3K/AKT inhibitor LY294002 could reverse this effect. Similarly, the Erk pathway is known to be involved in cell proliferation and differentiation, and has been linked to cancer development and progression. Our results suggest that DMC-HA can also inhibit this pathway, potentially contributing to its ability to inhibit EMT in GBM cells.

## CONCLUSIONS

These findings have important implications for the development of new treatments for GBM. By targeting multiple signaling pathways involved in EMT, DMC-HA may have a more potent and effective anti-cancer activity compared to drugs that target a single pathway. Furthermore, our study provides new insights into the mechanisms underlying the anti-cancer effects of DMC-HA, which may facilitate the development of more effective therapies for cancer patients. Overall, our results suggest that DMC-HA has great potential as a novel therapeutic agent for the treatment of cancer.
